# Prospective Study Reveals Host Microbial Determinants of Clinical Response to Fecal Microbiota Transplant Therapy in Type 2 Diabetes Patients

**DOI:** 10.3389/fcimb.2022.820367

**Published:** 2022-03-25

**Authors:** Dafa Ding, Huijuan Yong, Na You, Wei Lu, Xu Yang, Xiaolong Ye, Yayun Wang, Tingting Cai, Xiaoling Zheng, Hui Chen, Bota Cui, Faming Zhang, Xingyin Liu, Jian-Hua Mao, Yibing Lu, Hang Chang

**Affiliations:** ^1^ Department of Endocrinology, The Second Affiliated Hospital of Nanjing Medical University, Nanjing, China; ^2^ Key Laboratory of Modern Toxicology of Ministry of Education, School of Public Health, Nanjing Medical University, Nanjing, China; ^3^ Biological Systems and Engineering Division, Lawrence Berkeley National Laboratory, Berkeley, CA, United States; ^4^ Biomedical Data Science Center, Lawrence Berkeley National Laboratory, Berkeley, CA, United States; ^5^ Medical Center for Digestive Diseases, The Second Affiliated Hospital of Nanjing Medical University, Nanjing, China; ^6^ Department of Pathogen Biology-Microbiology Division, Key Laboratory of Pathogen of Jiangsu Province, Nanjing Medical University, Nanjing, China

**Keywords:** fecal microbiota transplantation, type 2 diabetes mellitus, therapeutic biomarker, prospective cohort study, gut microbiome

## Abstract

**Background:**

Increasing evidence shows that alterations in gut microbiome (GM) contribute to the development of type 2 diabetes mellitus (T2DM), and fecal microbiota transplantation (FMT) successfully treats various human diseases. However, the benefits of FMT therapy to T2DM patients remain unknown.

**Methods:**

We enrolled 17 patients with T2DM for nonblinded, one-armed intervention trial of FMT. A total of 20 healthy individuals were recruited as the baseline control. HbA1c% and metabolic parameter change were evaluated in 17 T2DM patients 12 weeks after they received FMT from healthy donors. The GM composition was characterized by 16S rRNA gene amplicon sequencing from fecal samples prior to and 12 weeks after FMT treatment.

**Results:**

We found that the GM of T2DM patients was reconstituted by FMT. We observed a statistically significant decrease in HbA1c% (from 7.565 ± 0.148 to 7.190 ± 0.210, p<0.01), blood glucose (from 8.483 ± 0.497 to 7.286 ± 0.454 mmol/L, p<0.01), and uric acid (from 309.4 ± 21.5 to 259.1 ± 15.8 µmol/L, p<0.01) while a significant increase in postprandial C-peptide (from 4.503 ± 0.600 to 5.471 ± 0.728 ng/ml, p<0.01) at 12 weeks after FMT. Closely evaluating the changes in these assays, we found individual variability in response to FMT treatment. Out of 17 T2DM patients, 11 were found to significantly improve T2DM symptoms. The FMT responders have significantly higher levels of the family *Rikenellaceae* and the genus *Anaerotruncus* (family *Ruminococcaceae*) in their pretreated fecal in comparison to nonresponders, which could predict the clinical response with an area under the curve of 0.83.

**Conclusion:**

Our findings suggest that certain T2DM patients can potentially benefit from FMT, and the pretreated abundance of *Rikenellaceae* and *Anaerotruncus* in the fecal of patients may serve as potential biomarkers for selecting T2DM patients to receive FMT.

## Introduction

Type 2 diabetes mellitus (T2DM) is a chronic disease characterized by high blood glucose levels (hyperglycemia), with rising global prevalence (Saeedi et al., 2019). T2DM develops primarily due to insulin resistance and inadequate insulin secretion. Successful management of T2DM frequently requires that hyperglycemia is simultaneously addressed. The use of pharmacological drugs as an addition to lifestyle measures is the cornerstone of T2DM treatment, and patients with T2DM who do not achieve their glycemic goal by taking an oral antidiabetic drug (OAD) alone eventually require additional therapy with insulin and other injecting drugs (Davies et al., 2018). Therefore, majority of people with T2DM have been treated by insulin injection, which is the most successful for controlling blood sugar (Home et al., 2014). However, episodic illness and aging can cause the body’s insulin resistance to increase; many patients still do not reach their glycated hemoglobin (HbA1c) targets. More importantly, such intensification of insulin regimens increases the risk of hypoglycemia and may lead to weight gain, which can increase cardiovascular risk and worsen weight-related comorbidities (Silver et al., 2018). Consequently, additional treatment is required over time.

The gut microbiome has recently emerged as an important factor associated with human diseases (Clemente et al., 2012; Cani, 2018). Moreover, many studies have demonstrated the changes in the gut microbiome of T2DM patients (Qin et al., 2012; Pedersen et al., 2016; Zhao et al., 2018), suggesting that gut microbiota plays an important role in T2DM. However, so far whether reconstitution of the gut microbiome, such as fecal microbiota transplantation (FMT) from a healthy donor, can serve as a potential treatment of T2DM patients has not been evaluated. Over the past decade, FMT has increasingly gained interest (de Groot et al., 2020) and has been successfully used to treat many human diseases such as inflammatory bowel disease (Qazi et al., 2017), obesity (Lee et al., 2019; Allegretti et al., 2020; Yu et al., 2020), metabolic syndrome (Kootte et al., 2017), and functional gastrointestinal disorders (Pamer, 2014; Cruz-Aguliar et al., 2019).

In this study, we conducted a prospective, nonblind, and single arm study to determine whether FMT therapy can bring any benefits to T2DM patients. Observation of individual variability in response to FMT further led us to investigate if differences in pretreated host gut microbiota could influence the beneficial effect of FMT in T2DM patients.

## Materials and Methods

### Study Design

**Figure 1:**
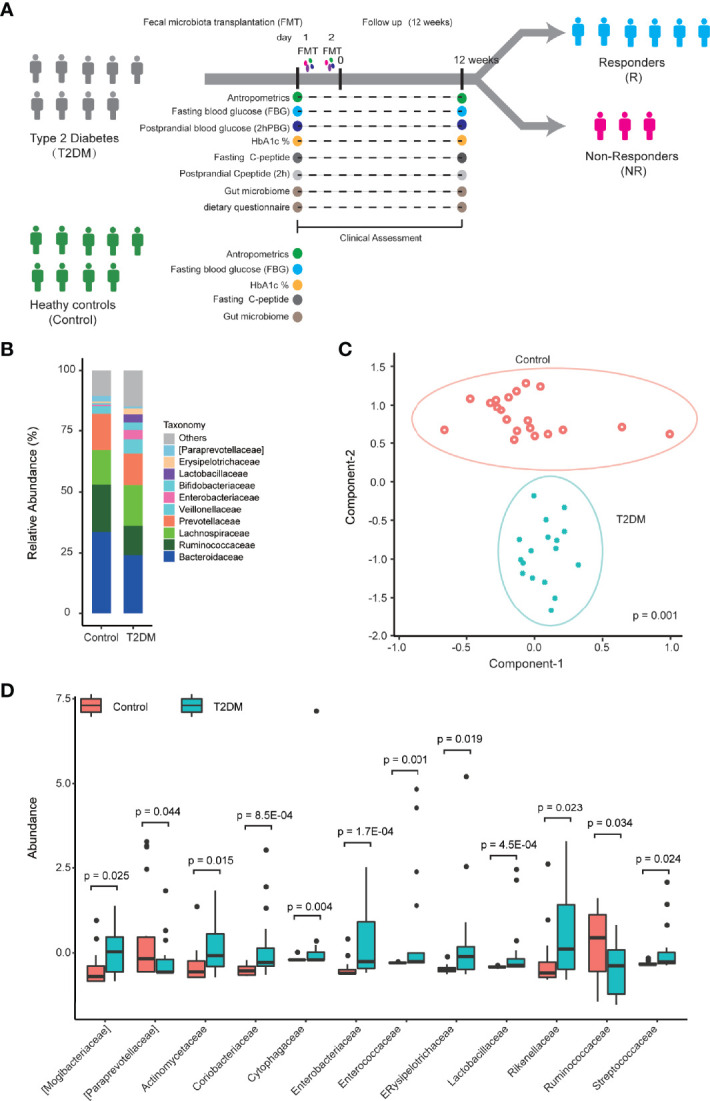
The composition of fecal microbiome is altered in T2DM patients. **(A)** A graphic illustration of the perspective cohort study design to evaluate clinical outcomes in T2DM patients following FMT. **(B)** The relative abundance of the major microbial families in T2DM patients and controls. **(C)** Maximally collapsing metric learning (MCML) analysis of family-level T2DM gut microbiomes. Seventeen T2DM samples (green cross) and 20 control samples (res open circle). The p-value was obtained from permutational multivariate analysis of variance (PERMANOVA). **(D)** Boxplot of relative abundance of 12 families that showed the significant difference between T2DM and control. The p-values were obtained by Mann–Whitney test. Boxes represent the median and interquartile ranges between the first and third quartiles. The dots represent outliers.

The current study is a nonblinded, one-armed intervention trial of FMT in T2DM patients inadequately controlled by metformin and daily insulin injections (HbA1c 6.5% and 8.5%) to evaluate the effect of FMT on glycemic control, body weight, and other metabolic parameters as well as safety and tolerability (**Figure 1A**). Before enrollment, all participants received diabetes education and anthropometric and metabolic assessments. Participants could keep their own diet but were asked to keep a nutritional diary to monitor daily caloric intake for seven days before clinical assessment. After two-day FMT treatment, participants were followed up for 12 weeks and were examined according to the study protocol at a ward of the hospital. Seventeen subjects were finally enrolled based on the inclusion and exclusion criteria discussed below. A total of 20 participants without diabetes served as the healthy control in the hospital physical examination center who were matched to participants with diabetes by sex, BMI, and other clinical parameters.

Inclusion criteria were as follows: (i) age between 18 and 71 years; (ii) T2DM diagnosis in the previous 12 months, as defined by the American Diabetes Association Criteria (American Diabetes, A., 2010); (iii) absence of systemic and metabolic disease other than T2DM, and absence of infection within the previous 3 months; (iv) absence of diet or medication that might interfere with glucose homeostasis, such as glucocorticoids or antibiotics in the previous 3 months; and (v) HbA1c lower than 8.5%.

Exclusion criteria were as follows (Zhao et al., 2018): (i) clinically significant major systemic disease, including malignancy; (ii) severe diabetic complications (diabetic retinopathy, diabetic neuropathy, diabetic nephropathy, and diabetic foot); (iii) continuous antibiotic use for >3 days within 3 months prior to enrollment, alcohol abuse, defined as >80 g/day in men and >40 g/day in women, and continuous use of weight-loss drug for > 1 month; (iv) severe organic diseases, including coronary heart disease, myocardial infarction, or cerebral apoplexy; (v) acute illnesses or current evidence of acute or chronic inflammatory or infective disease; and (vi) mental illness rendering the participants unable to understand the nature, scope, and possible consequences of the study.

The study was approved by the Ethical Committees of the second affiliated hospital of Nanjing Medical University, Nanjing, China (protocol ID: [2015]KY 044), and the trial was registered in the Chinese Clinical Trial Registry (ChiCTR-ONC-17011792). All participants gave written informed consent before enrollment. No changes to methods were made after trial commencement. The trial was concluded when all the participants had finalized their last visit.

### Fecal Microbiota Transplantation Procedure

All T2DM participants received FMT treatment twice (**Figure 1A**) by a novel technology named “Transendoscopic enteral tubing (TET)” that was used to insert a TET-tube (Nanjing FMT Medical Co., Ltd, China) into the participants’ mid-gut for the repeat FMTs same as the previous reports (Cui et al., 2015). The TET-tube was kept for the subsequent two days to deliver fecal microbiota. At FMT, 4 units of the bacterial suspension were injected into the mid-gut through the tube using 50-ml syringes. Donors were recruited according to the long-term criteria for donor selection at our center, including age, physiology, pathology, psychology, honesty, time, environment, and recipient state (Zhang et al., 2018; Ding et al., 2019). The improved methodology of FMT based on the automatic washing process (Zhang et al., 2020) and the related delivering consideration was named as washed microbiota transplantation (WMT) by the consensus statement from the FMT-standardization Study Group in 2019 (Fecal Microbiota Transplantation-standardization Study Group, 2020).

### Plasma and Feces Sample Collection

We collected plasma samples prior to and 12 weeks after FMT treatment. Blood samples were collected after 10 h of overnight fasting and 2 h after breakfast in a ward of the second affiliated hospital of Nanjing Medical University at each visit. All blood samples were set at room temperature for 30 min and then centrifuged at 3,000 x g for 20 min to obtain the serum. Feces samples were collected on the same day, snap-frozen in dry ice, and stored at −80°C until analysis (Pedersen et al., 2016).

### Blood Glucose Monitoring and Insulin Dose Adjustment

To conduct therapy on T2DM patients and improve their glucose homeostasis by FMT, we titrated the insulin dose to reduce hyperglycemia of patients according to the patients’ glucose levels (Tuttle et al., 2018). During the 12-week treatment period, patients were instructed to titrate their basal insulin analog dose or premix insulin analogs to achieve a pre-breakfast blood glucose level of 4.4 to 6.7 mmol/L and to titrate their bolus insulin dose to achieve a pre-lunch, pre-dinner, and bedtime blood glucose level of 4.4 to 6.7 mmol/L. Algorithms for titrating basal and bolus insulin doses to achieve target glycemic goals were provided as a general guideline (Strange, 2007). Investigators were allowed to use their judgment when recommending insulin adjustments based on their knowledge of the patient’s individual history. Patients were required to record daily glucose measurements and concomitant doses of basal and bolus insulin at specified time points to assess compliance with insulin dosing and to receive titration instructions.

### Definition of Responders and Nonresponders

On the basis of A1C reduction and the changes of fast blood glucose by FMT, patients were categorized into responders and nonresponders. There is no accepted criterion in the clinical cutoff point to divide patients into responders and nonresponders after 12 weeks of FMT treatment. Thus, we selected the criteria based on our clinical experiences and previous studies as follows: (1) A novel index called “A1c index” where the changes of HbA1c levels (ΔHbA1c) were adjusted by the baseline HbA1c levels (ΔHbA1c/baseline HbA1c) (Charpentier et al., 2001; Jones et al., 2014; Simioni et al., 2018) was used to assess the glycemic efficacy of FMT. Patients with A1c index ≤ -0.05 were termed as responders, while those with A1c index > -0.05 were termed as nonresponders. This cutoff value was the borderline where the changes of HbA1c levels become significant or nonsignificant. (2) Fast blood glucose (FBG) levels had decreased by ≥10% from the baseline within three months of FMT therapy, which were termed as responders. In all other cases, patients were nonresponders.

### Microbiome Analysis

Sequencing libraries were generated using Ion Plus Fragment Library Kit 48 rxns (Thermo Scientific) following the manufacturer’s recommendations. The library quality was assessed on the Qubit@ 2.0 Fluorometer (Thermo Scientific). At last, the library was sequenced on an Ion S5TM XL platform, and 400 bp/600 bp single-end reads were generated. High-quality reads were clustered into operational taxonomic units (OTUs) using QIIME (Quantitative Insights Into Microbial Ecology, V1.9.1) with an open-reference OTU picking process. Reads of 97% similarity to the reference database (Greengenes OTUs (16S) v13_8) were clustered into the same OTU. All samples were rarefied to 53,467 reads by random subtraction, which was the smallest sample size. Alpha diversity indices, including observed species, Chao1, Shannon, and Simpson were calculated by QIIME diversity analysis.

We first sought to characterize the gut microbiome in T2DM patients by 16S rRNA sequencing on a total of 37 fecal samples from 17 individuals with T2DM and 20 healthy controls using Illumina MiSeq. After removing low-quality reads and human DNA reads, a total of 4,308,192 high-quality reads remained (ranging from 53,467 to 94,639 reads per sample). Finally, each sample was rarefied to 53,467 reads that were clustered into OTUs at 97% similarity (**Supplementary Table 1**).

Mann–Whitney test (rank-sum or paired, as appropriate) was utilized to identify microbiome features that have significantly different abundance (p-value < 0.05) among control, responders, and nonresponders across timepoints (i.e., before FMT and after FMT). Maximally collapsing metric learning (MCML) analysis was adopted to show the separation of family-level gut microbiomes between T2DM and control samples, where ANOVA (PERMANOVA) is used for formal statistical test to investigate the statistically significant difference between groups (p-value < 0.05). MCML is an information-theory-based distance metric learning algorithm that aims to discovery the data pattern in N-dimensional spaces. It obtains a metric that minimizes the Kullback–Leibler divergence to separate samples within different groups. It was used to explore the microbiome data structure across groups. Spearman’s correlation was conducted to correlate the abundance of genera and clinical factors using both T2DM and control samples. Random forest analysis was used to 1) assess the association between microbial abundance at the genus level and the clinical factor, and 2) evaluate the effectiveness of preselected microbiome features for the prediction of clinical response to FMT with leave-one-out cross-validation and 1,000 trees.

### Statistical Analysis

Demographic, anthropometric, and clinical characteristics at baseline of the patients assigned to each group are presented (as mean ± SD). Differences in these characteristics between the healthy control and T2DM groups were compared using Mann–Whitney test (paired or rank-sum, as appropriate). The FMT treatment effect for laboratory measures (serum levels of different metabolites) was evaluated using an analysis of Mann–Whitney test (paired or rank-sum, as appropriate). The primary endpoint, HbA1c change, was evaluated from baseline (day 1) to week 12 visit (W12, trial day 90). Other prespecified efficacy endpoints included change from baseline in FPG and percent change in body weight at week 12. Change in systolic and diastolic BP and percent changes in fasting lipids were also evaluated.

All statistical analyses were performed using the R software (version 3.6.0) and Matlab (version 2012b), and the following packages were used: Stats (R, version 3.6.1), Random Forest (Matlab, Version 4.6-14), and Statistics Toolbox (Matlab, Version 8.1). A P < 0.05 was considered significant in all analysis. All statistical parameters can be found in the figure legends and tables.

## Results

### Gut Bacteria Associated With T2DM

Based on our patient selection criteria, 17 T2DM patients were finally enrolled into our perspective cohort study (ChiCTR-ONC-17011792) to assess clinical outcomes following FMT treatment (**Figure 1A**). A total of 17 T2DM patients had clinical symptoms for 1 year or more and were inadequately controlled with multiple daily insulin injections in combination with metformin treatment. Clinical assessment shows that these patients still had significantly higher levels of blood sugar and hemoglobin A1c (HbA1c) in comparison to 20 healthy controls (**Table 1**).

**Table 1:** Comparison of the baseline study parameters between healthy control and T2DM.

Parameter	Control (n = 20)	T2DM (n = 17)	p value
Age (years)	41.80 ± 2.09	57.18 ± 2.71	< 0.01
Gender			0.33
Male	12 (60%)	7 (41.2%)	
Female	8 (40%)	10 (58.8%)	
Duration of diabetes (years)	No	12	NA
Height (m)	171.4 ± 2.0	165.2 ± 1.9	0.03
Weight (kg)	67.65 ± 2.04	69.22 ± 2.33	0.61
BMI (kg/m^2^)	23.70 ± 0.70	25.45 ± 0.63	0.07
Blood pressure systolic (mmHg)	115.9 ± 3.8	127.6 ± 2.8	0.02
Blood pressure diastolic (mmHg)	71.10 ± 2.82	78.53 ± 2.25	0.05
Fasting glucose (mmol/L)	5.563 ± 0.114	8.483 ± 0.497	< 0.01
HbA1c (%)	5.685 ± 0.061	7.565 ± 0.148	< 0.01
Alanine transaminase, ALT (IU/L)	22.10 ± 2.80	24.49 ± 2.18	0.52
Aspartate aminotransferase, AST (IU/L)	21.30 ± 1.65	21.80 ± 2.26	0.86
Uric acid, UA (µmol/L)	319.5 ± 15.6	309.4 ± 21.5	0.70
Cholesterol: total (mmol/L)	5.316 ± 0.200	3.908 ± 0.274	<0.01
Cholesterol: triglycerides (mmol/L)	1.372 ± 0.182	2.244 ± 0.353	0.03
Cholesterol: HDL (mmol/L)	1.530 ± 0.061	0.963 ± 0.065	<0.01
Cholesterol: LDL (mmol/L)	3.177 ± 0.160	2.354 ± 0.217	<0.01
Blood urea nitrogen (mmol/L)	5.220 ± 0.291	6.375 ± 0.444	0.03
Serum creatinine concentration, SCr (µmol/L)	77.47 ± 3.42	64.74 ± 4.15	0.02

P values were obtained by Mann–Whitney rank-sum test.

Continuous variables are shown as the means (SD) and categorical variables as indicated. HbA1c of 5.685% converts to 39 mmol/mol, 7.565% to 56 mmol/mol. NA, Not applicable.

We first sought to characterize the gut microbiome in T2DM patients by 16S rRNA sequencing on a total of 37 fecal samples from 17 individuals with T2DM and 20 healthy controls using Illumina MiSeq. After removing low-quality reads and human DNA reads, a total of 4,308,192 high-quality reads remained (ranging from 53,467 to 94,639 reads per sample). Finally, each sample was rarefied to 53,467 reads that were clustered into OTUs at 97% similarity (**Supplementary Table 1**). Although there is no significant difference in alpha diversity measured by Chao, Shannon, Simpson, and the ACE index (**Supplementary Figures 1A–D**), we observed significant taxonomic differences in fecal microbiota between T2DM patients and controls (**Figures 1B–D** and **Supplementary Figure 2**; **Supplementary Table 2**). The relative abundances of taxa classified to the family level have significant changes in comparison to healthy controls (**Figure 1B**). The T2DM and control samples showed clear separation by maximally collapsing metric learning (MCML) analysis of all families (**Figure 1C**). Moreover, we found a significant increase in the abundance of the families *Rikenellaceae*, *Lactobacillaceae*, *Enterobacteriaceae*, *Coriobacteriaceae*, *Enterococcaceae*, *Streptococcaceae*, *Cytophagaceae*, *Actinomycetaceae*, and *Erysipelotrichaceae* and a significant decrease in the abundance of the family *Ruminococcaceae* in T2DM samples (**Figure 1D**). At the genus level, many genera showed significantly different abundances between T2DM and controls. The genera *Blautia* and *Lactobacillus* are significantly positively correlated with T2DM, while the genus *Faecalibacterium* is significantly negatively correlated with T2DM (**Supplementary Figure 3**), which are consistent with previous studies (Egshatyan et al., 2016; Remely et al., 2016; Lippert et al., 2017; Sedighi et al., 2017).

### Links Between the Gut Microbiome and Clinical Factors of T2DM

**Figure 2:**
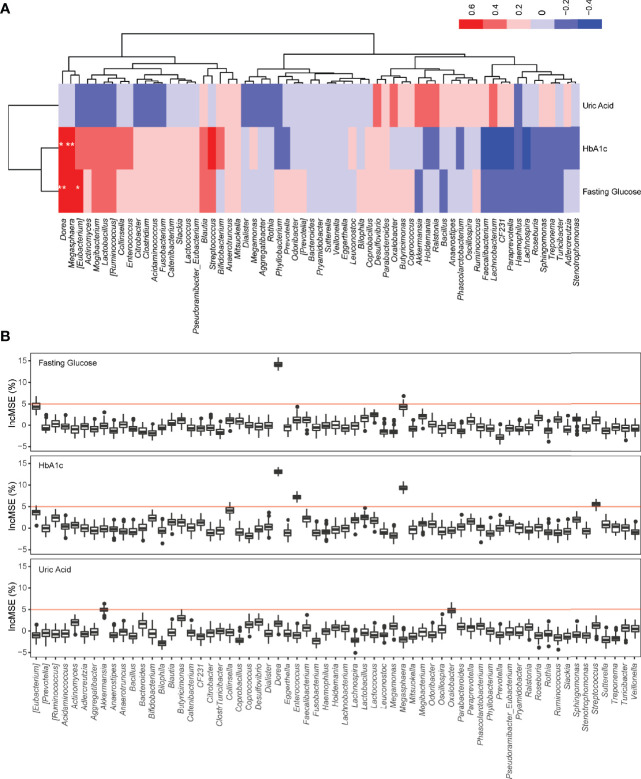
The relationship between the genus-level microbial abundance and clinical factors. **(A)** Heatmap of the Spearman’s correlation coefficients between the abundance of genera and clinical factors using both T2DM and control samples. *p < 0.05; **p < 0.01. **(B)** Random forest analysis to assess the association between microbial abundance at the genus level and clinical factor. Significant level is indicated in red line (p < 0.05).

To further determine the links between the gut microbiome and T2DM, we correlated the taxonomic profile to clinical data related to T2DM. At the genus level, the abundance of *Dorea* is significantly positively correlated with the levels of fasting glucose and hemoglobin A1c (HbA1c) in blood, and the abundance of *Megasphaera* is also significantly positively correlated with the levels of HbA1c (**Figure 2A**). At the family level, the abundance of *Enterobacteriaceae* is significantly positively correlated with the levels of fasting glucose, while none of the families is significantly correlated with the levels of HbA1c (**Supplementary Figure 3A**). We did not find any significant correlation between the levels of uric acid and abundance at the genus and family levels (**Figure 2A**; **Supplementary Figure 3A**). Random forest analysis further confirmed these observations (**Figure 2B**; **Supplementary Figure 3B**). A recent study also shows a positive correlation between the abundance of *Dorea* and fasting blood glucose (Adeshirlarijaney and Gewirtz, 2020a).

### Clinical Improvement of FMT in a Subset of T2DM Patients

**Figure 3:**
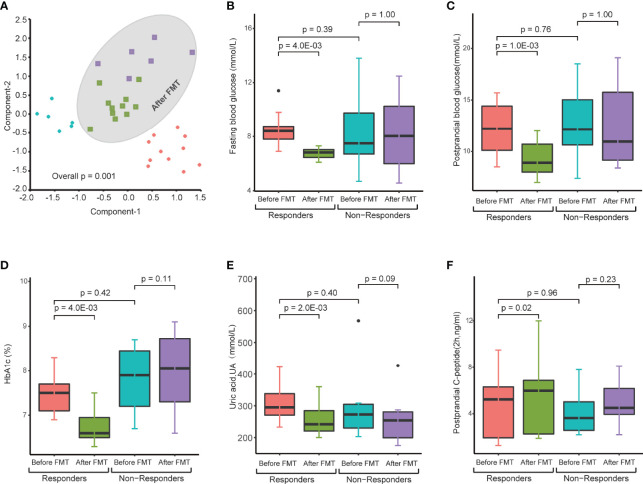
Changes in gut microbiota and clinical factors after FMT treatment. **(A)** PCA of gut microbiomes of T2DM patients before and after FMT treatment. The microbiome samples after FMT (inside green circle) are separated from pretreated microbiome samples (outside green circle). Clear separation of pretreated microbiome samples between responders (red diamonds) and nonresponders (blue diamonds). **(B–F)** These boxplots show changes in clinical factors before and after FMT treatment in responders and nonresponders. The p-values within the responder and nonresponder groups were obtained by Mann–Whitney paired test, and p-values across groups were obtained by Mann–Whitney rank-sum test.

A profound change in the gut microbiome of T2DM patients and significant correlation between specific microbes and clinical parameters suggest that the restoration of a healthy gut microbiota could lead to a potential clinical benefit. Therefore, we next determined the clinical impact of treatment with FMT from healthy donors on T2DM patients. All 17 T2DM patients received two FMTs (4 units of bacterial suspension for one FMT) (**Figure 2A**) through a mid-gut TET technique (Cui et al., 2015). 16S rRNA sequencing analysis verified that the gut microbiome of T2DM patients was reconstituted by FMT (**Figure 3A**). Twelve weeks post FMT treatment, the clinical outcomes of all patients were evaluated by measuring the levels of glycated hemoglobin (HbA1c) and other clinical markers related to T2DM. Overall, the symptoms of T2DM patients were significantly reduced by FMT (**Supplementary Table 3**), but a large variation in the level of fasting blood glucose and HbA1c among individual patients before and after FMT treatment was observed (**Supplementary Figure 4**). We assessed FMT treatment efficiency by defining a patient as a responder according to clinical criteria (Charpentier et al., 2001; Jones et al., 2014; Simioni et al., 2018), and found that 11 of the 17 T2DM patients responded to FMT treatment (**Figures 3B–F** and **Table 2**). In 11 responders, the levels of both fasting and postprandial glucose, HbA1c, and uric acid significantly decreased, while the levels of postprandial C-peptide significantly increased (**Figures 3B–F** and **Table 2**). In contrast, in 6 nonresponders, these clinical parameters did not change (**Figures 3B–F** and **Table 2**). Although we observed statistically significant reduction in HbA1c, the HbA1c level is still above clinical criteria (e.g., <6.5%). Nevertheless, these results indicate that some T2DM patients will significantly benefit from FMT therapy, and the effect of FMT on HbA1c reduction requires further evaluation/confirmation from larger studies.

**Table 2:** Changes in the study parameters before and after FMT treatment in responders and nonresponders.

	Responders (n=11)			Nonresponders (n=6)			
Parameter	Before FMT	After FMT (3months)	p value	Before FMT		After FMT (3months)	p value
Age (years)	55.73 ± 3.79	55.73 ± 3.79	NA	59.83 ± 3.43		59.83 ± 3.43	NA
Gender			NA				NA
Male	4 (26.7%)	4 (26.7%)		3 (50%)		3 (50%)	
Female	11 (73.3%)	11 (73.3%)		3 (50%)		3 (50%)	
Duration of diabetes (years)	12.36 ± 2.52	12.36 ± 2.52	NA	12.33 ± 3.37		12.33 ± 3.37	NA
Height (m)	167.5 ± 2.5	167.5 ± 2.5	NA	161.2 ± 1.8		161.2 ± 1.8	NA
Weight (Kg)	70.25 ± 3.59	69.45 ± 3.63	0.08	67.33 ± 0.84		68.00 ± 1.10	0.17
BMI (Kg/m2)	24.99 ± 0.88	24.55 ± 0.92	0.06	26.31 ± 0.68		26.56 ± 0.79	0.20
Blood pressure systolic (mmHg)	128.5 ± 3.8	126.4 ± 1.8	0.59	125.8 ± 4.2		126.2 ± 4.7	1.00
Blood pressure diastolic (mmHg)	78.64 ± 3.43	74.09 ± 2.22	0.08	78.33 ± 1.67		81.17 ± 0.83	0.18
Fasting glucose (mmol/L)	8.53 ± 0.37	6.77 ± 0.12	<0.01	8.40 ± 1.32		8.23 ± 1.24	1.00
Postprandial glucose (2h, mmol/L)	12.19 ± 0.75	9.28 ± 0.506	<0.01	12.72 ± 1.62		12.55 ± 1.83	1.00
HbA1c (%)	7.44 ± 0.14	6.77 ± 0.12	<0.01	7.80 ± 0.33		7.97 ± 0.40	0.12
Alanine Transaminase, ALT (IU/L)	25.78 ± 2.93	22.75 ± 2.37	0.08	22.13 ± 3.15		20.47 ± 3.16	0.31
Aspartate Aminotransferase, AST (IU/L)	22.82 ± 3.00	23.74 ± 3.07	0.57	19.93 ± 3.50		21.87 ± 3.95	0.09
Uric acid, UA (µmol/L)	310.2 ± 18.2	256.6 ± 15.5	<0.01	307.8 ± 54.6		263.5 ± 37.3	0.09
Cholesterol: total (mmol/L)	3.56 ± 0.34	3.24 ± 0.35	0.28	4.55 ± 0.38		4.70 ± 0.24	0.56
Cholesterol: triglycerides (mmol/L)	2.21 ± 0.44	1.77 ± 0.380	0.03	2.31 ± 0.64		2.26 ± 0.57	0.84
Cholesterol: HDL (mmol/L)	0.90 ± 0.06	0.88 ± 0.08	0.42	1.08 ± 0.14		0.95 ± 0.10	0.16
Cholesterol: LDL (mmol/L)	2.07 ± 0.29	1.82 ± 0.31	0.21	2.88 ± 0.20		3.42 ± 0.20	0.03
Blood urea nitrogen (mmol/L)	6.02 ± 0.42	5.92 ± 0.54	1.00	7.03 ± 0.10		5.82 ± 0.33	0.17
Serum creatinine concentration, SCr (µmol/L)	62.87 ± 5.28	61.46 ± 3.56	0.70	68.15 ± 7.12		67.05 ± 6.84	0.84
Fasting C-peptide (ng/ml)	2.39 ± 0.48	2.71 ± 0.40	0.24	2.16 ± 0.50		2.35 ± 0.50	0.22
Postprandial C-peptide (2h, ng/ml)	4.69 ± 0.82	5.75 ± 1.04	0.02	4.16 ± 0.87		4.97 ± 0.86	0.22

P values were obtained by Mann–Whitney paired test.

Continuous variables are shown as the means (SD) and categorical variables as indicated.

HbA1c of 7.44% converts to 58 mmol/mol, 6.77% to 50 mmol/mol, 7.8% to 62 mmol/mol, and 7.97% to 64 mmol/mol. NA, Not applicable.

### Influence of Pretreated Gut Microbiome on the Response to FMT

**Figure 4:**
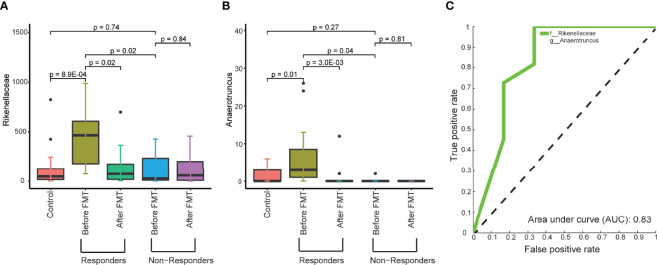
The microbial abundance in pretreated fecal samples related to the response to FMT treatment. **(A, B)** Boxplots show the abundance of the family *Rikenellaceae* **(A)** and genus *Anaerotruncus* **(B)** in the fecal samples from control, responders, and nonresponders before and after FMT treatment. **(C)** ROC of the model trained to predict the response to FMT treatment based on the abundance of the family *Rikenellaceae* and the genus *Anaerotruncus* in pretreated fecal samples. The p-values within the responder and nonresponder groups were obtained by Mann–Whitney paired test, and p-values across groups were obtained by Mann–Whitney rank-sum test.

We did not find any pretreated clinical factors associated with the response to FMT treatment (**Supplementary Table 4**). Therefore, the difference in the response to FMT treatment led us to explore the possibility that the pretreated gut microbiome of T2DM patients affects the outcome of FMT treatment. To do this, we compared the composition of gut microbiome between responders and nonresponders. We identified that significantly higher relative abundances of the family *Rikenellaceae* and the genus *Anaerotruncus* belong to the family *Ruminococcaceae* in responders compared to nonresponders (**Figure 4A** and **Supplementary Table 5**). Interestingly, the pretreated and post-treated abundances of *Rikenellaceae* and *Anaerotruncus* in nonresponders are similar to the level in healthy control. In contrast, in responders, the pretreated abundance of *Rikenellaceae* and *Anaerotruncus* is significantly higher than in healthy control, and FMT treatment adjusted their abundance to a healthy level (**Figures 4A, B**). This suggests that the abundance of *Rikenellaceae* and *Anaerotruncus* makes causal contribution to T2DM. Furthermore, random forest classification based on the relative abundant level of *Rikenellaceae* and *Anaerotruncus* led to the predictive accuracy of around 82.4% with an AUC of around 0.83 (**Figure 4C**). Our results suggest that the pretreated abundance of gut microbiota in T2DM patients determines the clinical response to FMT.

## Discussion

Compelling evidence shows that gut microbiome plays a role in the development of T2DM. Therefore, the reconstitution of gut microbiome can be an exciting avenue for possible novel treatment for T2DM patients. Although the sample size of our study was relatively small, our results provide strong evidence that modifying gut microbiota by FMT from a healthy donor can improve clinical outcomes of certain T2DM patients. However, like other therapies, at the end of our trial, we found that the FMT treatment did not benefit all patients, and the therapeutic effects of FMT were largely determined by the baseline gut microbiota composition. Additionally, no significant adverse events or gastrointestinal symptoms were found to be related to the FMT treatment, consistent with previous reports that a route of TET can offer a safe FMT procedure for outpatients (Zhang et al., 2018; Ding et al., 2019). These safety data are critical since FMT has garnered attention as a potential therapeutic option to improve diabetes.

Experimental studies and human clinical trials have shown the effects of various prebiotic and probiotic strains and their potential efficacy in ameliorating T2DM. In particular, they seem to confer beneficial effects by reversing dysbiosis and restoring the gut functional integrity (Adeshirlarijaney and Gewirtz, 2020b). Similarly, 6–12 weeks administration of *Lactobacillus acidophilus*, *Lactobacillus bulgaricus*, *Streptococcus thermophiles*, and/or *Bifidobacterium lactis* is effective in improving glycemic control in adults with T2DM (Gurung et al., 2020). However, many human association studies between gut microbiome and T2DM did not reach singular consensus implicating a particular set of bacteria with T2DM, suggesting that T2DM patients’ microbiomes are heterogenous. Therefore, it is not surprising to find that our study identified the significant association of many microbiota with T2DM, but only a small proportion of them overlap with previous studies (Egshatyan et al., 2016; Remely et al., 2016; Lippert et al., 2017; Sedighi et al., 2017).

In this study, we interestingly found that the transplantation of the fecal microbiota from healthy donors is sufficient to improve the glucose metabolism of T2DM, such as FBG, PBG, and HbA1c. Importantly, we also showed that FMT significantly increased the levels of postprandial C-peptide. This is similar to previous animal and human clinic reports, which showed that FMT improved the insulin resistance and attenuated pancreatic islet β-cell destruction (Kootte et al., 2017; Wang et al., 2019; de Groot et al., 2020). However, we did not observe weight loss after FMT treatment in T2DM. Also, we observed a high heterogeneous response to FMT in glucose homeostasis and insulin sensitivity. Only about 65% of T2DM patients benefit from FMT therapy. Although the reduction of HbA1c is statistically significant, its clinical value still requires further evaluation. Nevertheless, our findings suggest to develop the personalized treatment for T2DM patients based on their baseline microbial composition.

The potential effect of FMT on human glucose homeostasis and insulin sensitivity has already been established. FMT using a lean donor is sufficient to improve glucose homeostasis in the obese, which is associated with change in gut microbiota (Kootte et al., 2017; Rinott et al., 2021). The study also documented a small decline in HbA1c at 6 weeks after FMT. The minor improvement in HbA1c at 12 weeks was reported in another FMT-TRIM double-blind placebo-controlled pilot trial (Yu et al., 2020). In this study, we specifically observed a significant decline in HbA1c in T2DM after FMT treatment. Due to the fact that 65% of T2DM patients benefit from FMT therapy, the change of HbA1c responses to FMT may be attributed to complex host–recipient dynamic. Similar to previous FMT studies, allogenic FMT using metabolic syndrome donors decreases insulin sensitivity in the metabolic syndrome recipient. In contrast, post-Roux-en-Y gastric bypass donors’ FMT increases in peripheral insulin sensitivity (de Groot et al., 2020). This study suggested that the metabolic traits of allogenic FMT donors may drive the recipient metabolic response. With respect to the metabolic phenotype, FMT resulted in a significant decline in uric acid in the response recipients in our study. More interestingly, recent research reports that FMT stabled residual beta cell function in subjects with new-onset T1D (de Groot et al., 2021), which may further support the benefits of FMT therapy to diabetes patients. The findings suggested that the healthy phenotype and gut microbiota were at least partially transmissible, and that the “good” microbiome dominates the gut and attenuates the T2DM microbiome’s effects on glucose metabolism and insulin sensitivity.

Regulation of the intestinal microbial composition and function by FMT may only partially affect the intrinsic and complex pathophysiology of T2DM. In our study, we observed that responders had higher relative abundances of the family *Rikenellaceae* and the genus *Anaerotruncus* in pretreated fecal, while nonresponders had a similar level compared to healthy controls. After FMT treatment, within the responders, the abundance of the family *Rikenellaceae* and the genus *Anaerotruncus* was restored to a healthy level. In contrast, the abundance of the family *Rikenellaceae* and the genus *Anaerotruncus* has no change in nonresponders. These findings suggest that the family *Rikenellaceae* and the genus *Anaerotruncus* could play a causative role in T2DM, which needs further confirmation using animal models. For example, in the future, we will use metagenomic data analysis to further verify our results and then design specific media for the isolation of key species. Moreover, additional study can use germ-free mice inoculated with the key species identified from *Rikenellaceae* and/or *Anaerotruncus* to evaluate the causative impact of these microbials on T2DM in mice. If true, the animal models can be used to address whether special removal of these microbials can rescue the phenotype. These studies will provide evidence for clinical evaluation of special killing of *Rikenellaceae* and *Anaerotruncus* as a treatment option for T2DM patients.

Finally, we determined if fecal microbiota composition at baseline would be able to predict the responder or nonresponder status upon FMT with a good prediction (receiver operating characteristic [ROC] AUC 0.83). The primary strength of our study is to identify a potential therapeutic biomarker (the pretreated abundance of the family *Rikenellaceae* and the genus *Anaerotruncus*) that predicts the clinical response to FMT treatment for T2DM patients. Interestingly, *Rikenellaceae* has been reported to be enriched in diabetes and obesity (Kim et al., 2012; Gomez-Arango et al., 2016), which can modulate inflammatory responses and exert a pro-inflammatory effect in inflammatory bowel disease (IBD) (Loh and Blaut, 2012), contributing ultimately to metabolic dysfunction. Moreover, the genus *Anaerotruncus* has significant correlations with several metabolic parameters (Everard et al., 2011; Hasan et al., 2018), such as glucose intolerance, gut permeability, plasma triglyceride content, and muscle lipid content in Chinese, which may explain the improved glucose homeostasis in the responder patients. It is plausible that the metabolic effect of FMT in the T2DM was partly mediated through the persistent reduction of these bacteria by FMT during the glucose homeostasis. Moreover, we conclude that for future interventions, determining the baseline fecal microbiota composition might aid in predicting the efficacy of FMT treatment.

A few limitations of our study include the following: (1) a relatively small size of cohort without a control group; (2) lack of multiple time point research and long-term clinical effect since we only measured at 12 weeks post FMT treatment; (3) lack of underlying mechanism for response to FMT treatment; (4) for ethical and technical reasons, gut microbiome was assessed in fecal matter and not in bioactive sites along the gastrointestinal tract; and (5) the identified potential therapeutic biomarker needed further validation in independent cohorts. However, our findings pave a new way and raise a promising hope to utilize FMT to improve clinical outcomes of targeted T2DM patients.

In spite of the clinically evident effectiveness and safety of FMT provided by both our study and others, clinicians and researchers are compelled to find substitutes for FMT owing to the risk of disease transmission between the donor and the recipient, patients’ acceptance, undesirable outcomes, and the uncertain impacts on the recipient’s immune system. Aside from standardization of donor screening and clear protocols for adverse events monitoring, FMT registry should be set up to collect long-term data and follow-up outcomes and complications. Furthermore, we have gained much knowledge about the bacterial population in the human intestine over the last few years; however, little is known about the viral or fungal composition in the gut and even the function of intestinal bacteria. Additionally, another uncertainty of FMT is the highly dynamic composition of live microbiota, which is sensitive to external factors such as diet and drugs. Accordingly, future research should focus on identifying gut microbiota, defining their function, and further manipulating gut microbiota more precisely. In the near future, we will look forward to personalized FMT for different patients and conditions according to varied host and disease genotypes/phenotypes.

In conclusion, a gut microbiota biomarker can be used to predict the response to FMT treatment for T2DM patients. Our findings require further clinical evaluations in a large cohort across multiple hospitals, and future studies will focus on identifying active components of FMT to develop personalized approaches to treat T2DM and understanding mechanisms by which a gut microbiota biomarker may determine the outcome of FMT treatment.

## Data Availability Statement

The datasets presented in this study can be found in the Sequence Read Achieve (SRA) at the National Center for Biotechnology Information (NCBI) repository, accession number PRJNA626062.

## Ethics Statement

The studies involving human participants were reviewed and approved by the ethical committees of the second affiliated hospital of Nanjing Medical University, Nanjing, China (protocol ID: [2015]KY 044). The patients/participants provided their written informed consent to participate in this study.

## Author Contributions

HY, NY, TC, YW, XLY, XZ, HuC, DFD, YL, and WL recruited cohort individuals, performed the clinical study, and collected the clinic data. YW, XLY, XZ and HaC processed the samples. HaC, DFD, XY and XYL analyzed the data and all the results. XY generated all the final figures. BC and FZ performed and provided the fecal microbiota transplantation technical assistance. DFD, YL, and HaC designed and supervised the study. J-HM, DFD, and HaC wrote the manuscript. All the authors edited the manuscript and approved the final manuscript.

## Funding

This work was supported by the grants from the Major Research and Development Project of Jiangsu (BE2016800), Jiangsu Youth Medical Talents Project (QNRC2016674), Nanjing Science Technology Plan Project (201715015), Scientific research projects of Jiangsu Provincial Health and Health Commission (ZDB2020034, M2021056).

## Conflict of Interest

The authors declare that the research was conducted in the absence of any commercial or financial relationships that could be construed as a potential conflict of interest.

## Publisher’s Note

All claims expressed in this article are solely those of the authors and do not necessarily represent those of their affiliated organizations, or those of the publisher, the editors and the reviewers. Any product that may be evaluated in this article, or claim that may be made by its manufacturer, is not guaranteed or endorsed by the publisher.
