# Supplementary material for: Prospective Study Reveals Host Microbial Determinants of Clinical Response to Fecal Microbiota Transplant Therapy in Type 2 Diabetes Patients
Source: Front Cell Infect Microbiol. 2022 Mar 25;12:820367. doi: 10.3389/fcimb.2022.820367 (PMC8990819; doi:10.3389/fcimb.2022.820367)
Supplement: Supplementary file 1 [file Image_1.pdf]

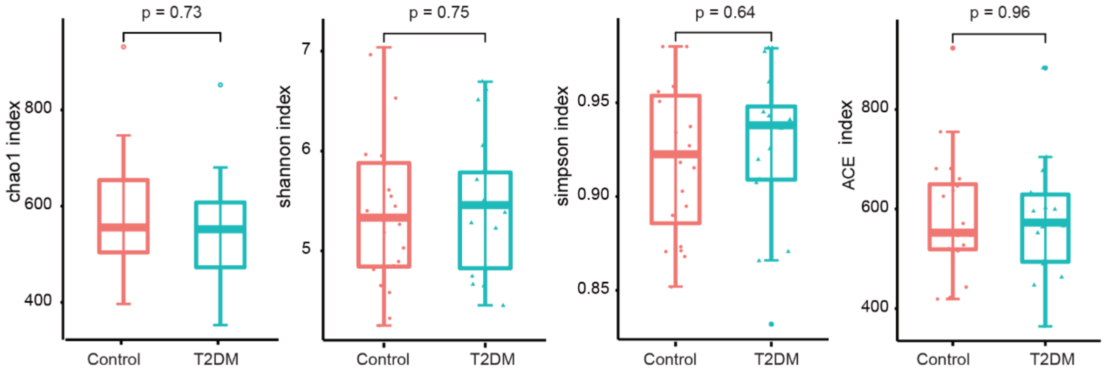

Supplementary Figure 1. No difference in alpha-diversity (Chao, Shannon, Simpson and ACE index) between control and T2DM samples. The p-values were obtained by Mann-Whitney rank-sum test. Boxes represent the median and interquartile ranges between the first and third quartiles. The dots represent outliers.

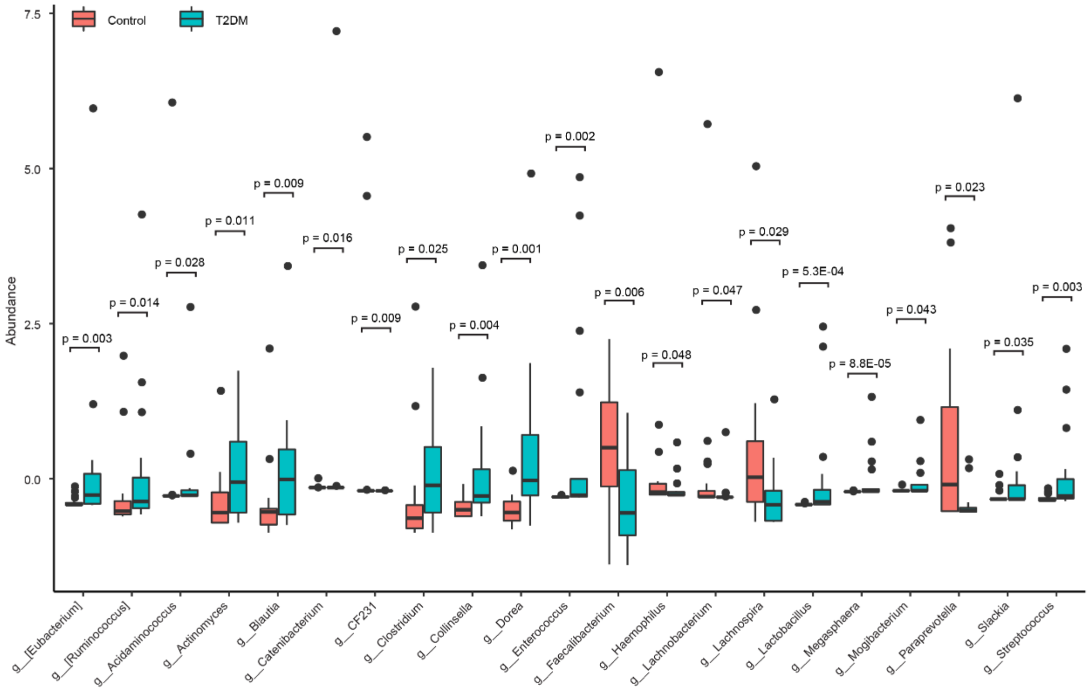

Supplementary Figure 2. Box-plot of relative abundance of 21 genera that showed the significant difference between T2DM and control. The p-values were obtained by Mann-Whitney rank-sum test. Boxes represent the median and interquartile ranges between the first and third quartiles. The dots represent outliers.

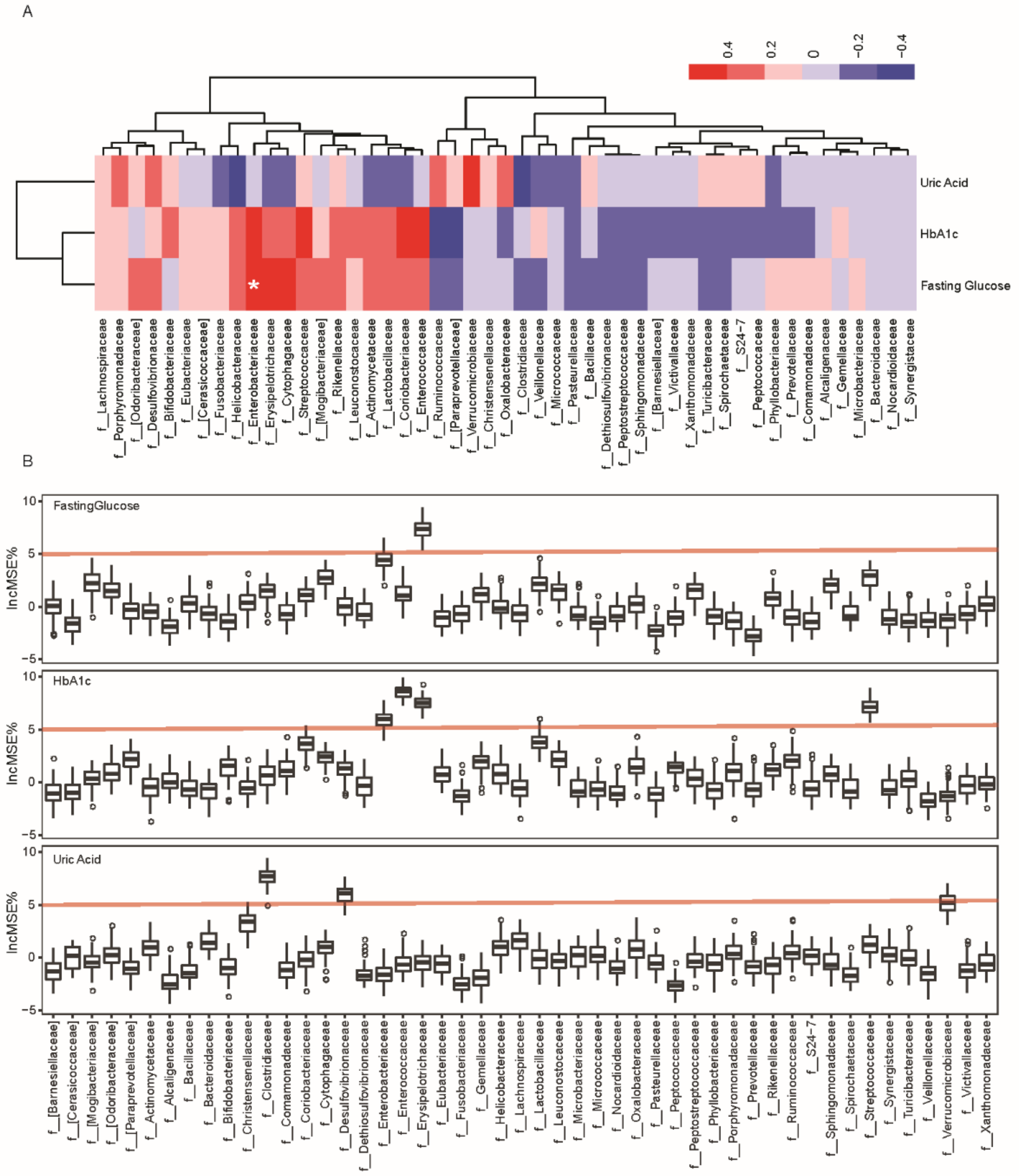

Supplementary Figure 3. The relationship between the family-level bacterial abundance and clinical factors. A. Heatmap of the Spearman's correlation coefficients between the family-level bacterial abundance and clinical factors using both T2DM and control samples. *p<0.05. **B.** Random forest analysis to assess the association between the family-level bacterial abundance and clinical factors. Significant level is indicated in red line.

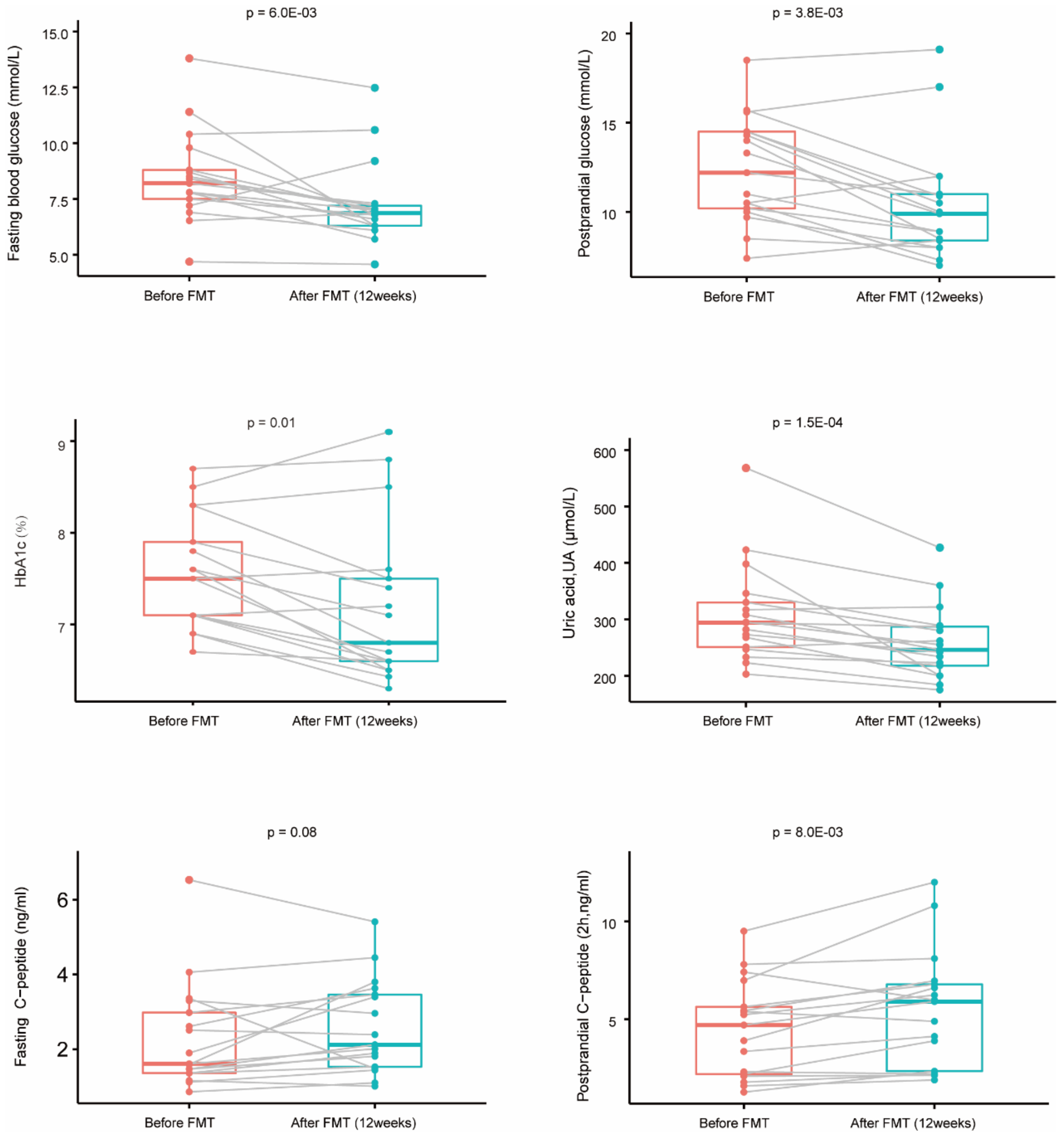

Supplementary Figure 4. Changes in clinical factors after FMT treatment. The p-values were obtained by Mann-Whitney paired test. Boxes represent the median and interquartile ranges between the first and third quartiles.
