# Supplementary material for: Prospective Study Reveals Host Microbial Determinants of Clinical Response to Fecal Microbiota Transplant Therapy in Type 2 Diabetes Patients
Source: Front Cell Infect Microbiol. 2022 Mar 25;12:820367. doi: 10.3389/fcimb.2022.820367 (PMC8990819; doi:10.3389/fcimb.2022.820367)
Supplement: Supplementary file 4 [file Table_3.docx]

| **Supplementary Table 3. Changes in the study parameters of T2DM between before and after FMT treatment. P values were obtained by Mann-Whitney paired test.** | | | |
| --- | --- | --- | --- |
| Parameters | Before FMT (n=17) | After FMT (n=17) | p value |
| Age (years) | 55.73 ± 2.62 | 55.73 ± 2.62 | NA |
| Gender |  |  | NA |
| Male | 7 (41.2%) | 7 (41.2%) |  |
| Female | 10 (58.8%) | 10 (58.8%) |  |
| Duration of diabetes (years) | 12.36 ± 1.74 | 12.36 ± 1.74 | NA |
| Height (m) | 165.2 ± 1.9 | 165.2 ± 1.9 | NA |
| Weight (Kg) | 69.22 ± 2.33 | 68.94 ± 2.34 | 0.41 |
| BMI (Kg/m2) | 25.45 ± 0.63 | 25.26 ± 0.68 | 0.13 |
| Blood pressure systolic (mmHg) | 127.6 ± 2.8 | 126.3 ± 1.9 | 0.26 |
| Blood pressure diastolic (mmHg) | 78.53 ± 2.25 | 76.59 ± 1.67 | 0.14 |
| Fasting glucose (mmol/L) | 8.483 ± 0.497 | 7.286 ± 0.454 | **<0.01** |
| Postprandial glucose (mmol/L) | 12.38 ± 0.72 | 10.44 ± 0.79 | **< 0.01** |
| HbA1c (%) | 7.565 ± 0.148 | 7.190 ± 0.210 | **<0.01** |
| Alanine Transaminase, ALT (IU/L) | 24.49 ± 2.18 | 21.94 ±1.86 | **0.03** |
| Aspartate Aminotransferase, AST (IU/L) | 21.80 ± 2.26 | 23.08 ± 2.36 | 0.18 |
| Uric acid, UA (µmol/L) | 309.4 ± 21.5 | 259.1 ± 15.8 | **< 0.01** |
| Cholesterol: total (mmol/L) | 3.908 ± 0.274 | 3.753 ± 0.292 | 0.45 |
| Cholesterol: triglycerides (mmol/L) | 2.211 ± 0.444 | 1.944 ± 0.313 | 0.08 |
| Cholesterol: HDL (mmol/L) | 0.9629 ± 0.0645 | 0.8994 ± 0.0598 | 0.16 |
| Cholesterol: LDL (mmol/L) | 2.354 ± 0.217 | 2.381 ± 0.282 | 0.87 |
| Blood urea nitrogen (mmol/L) | 6.375 ± 0.444 | 5.883 ± 0.359 | 0.31 |
| Serum creatinine concentration, SCr (µmol/L) | 64.74 ± 4.15 | 63.44 ± 3.28 | 0.52 |
| Fasting C-peptide (ng/ml) | 2.309 ± 0.347 | 2.582 ± 0.307 | 0.24 |
| Postprandial C-peptide (2h, ng/ml) | 4.503 ± 0.600 | 5.471 ± 0.728 | **<0.01** |

Continuous variables are shown as the means (SD) and categorical variables as indicated. HbA1c of 7.565 % converts to 59 mmol/mol, 7.190% to 55 mmol/mol.
