# Supplementary material for: Prospective Study Reveals Host Microbial Determinants of Clinical Response to Fecal Microbiota Transplant Therapy in Type 2 Diabetes Patients
Source: Front Cell Infect Microbiol. 2022 Mar 25;12:820367. doi: 10.3389/fcimb.2022.820367 (PMC8990819; doi:10.3389/fcimb.2022.820367)
Supplement: Supplementary file 5 [file Table_4.docx]

| **Supplementary Table 4. Comparison of the study parameters between responders and non-responders at baseline. P values were obtained by Mann-Whitney rank-sum test.** | | | |
| --- | --- | --- | --- |
| Parameter | Responders (n=11) | Non-Responders (n=6) | p value |
| Age (years) | 55.73 ±3.793 | 59.83 ±3.429 | 0.49 |
| Gender |  |  | 0.64 |
| Male | 4 (36.4%) | 3 (50%) |  |
| Female | 7 (63.6%) | 3 (50%) |  |
| Duration of diabetes (years) | 12.36 ± 2.52 | 12.33 ± 3.37 | 0.99 |
| Height (m) | 167.5 ± 2.5 | 161.2 ± 1.8 | 0.11 |
| Weight (Kg) | 70.25 ± 3.59 | 67.33 ± 0.843 | 0.57 |
| BMII (kg/m2) | 25.61 ± 0.65 | 26.08 ± 0.78 | 0.71 |
| Blood pressure systolic (mmHg) | 129.4 ± 4.0 | 125.8 ± 4.2 | 0.57 |
| Blood pressure diastolic (mmHg) | 78.64 ± 3.43 | 78.33 ± 1.67 | 0.95 |
| Fasting glucose (mmol/L) | 8.527 ± 0.368 | 8.402 ± 1.320 | 0.91 |
| Postprandial glucose (2h, mmol/L) | 12.19 ± 0.75 | 12.72 ± 1.62 | 0.74 |
| HbA1c (%) | 7.436 ± 0.137 | 7.800 ± 0.334 | 0.25 |
| Alanine Transaminase, ALT (IU/L) | 25.78 ± 2.93 | 22.13 ± 3.15 | 0.44 |
| Aspartate Aminotransferase, AST (IU/L) | 22.82 ± 3.00 | 19.93 ± 3.50 | 0.56 |
| Uric acid, UA (µmol/L) | 310.2 ± 18.2 | 307.8 ± 54.6 | 0.96 |
| Cholesterol: total (mmol/L) | 3.558 ± 0.335 | 4.550 ± 0.379 | 0.08 |
| Cholesterol: triglycerides (mmol/L) | 2.211 ± 0.444 | 2.305 ± 0.635 | 0.90 |
| Cholesterol: HDL (mmol/L) | 0.902 ± 0.063 | 1.075 ± 0.138 | 0.21 |
| Cholesterol: LDL (mmol/L) | 2.066 ± 0.287 | 2.880 ± 0.196 | 0.07 |
| Blood urea nitrogen (mmol/L) | 6.016 ± 0.423 | 7.033 ± 0.998 | 0.29 |
| Serum creatinine concentration, SCr (µmol/L) | 62.87 ± 5.28 | 68.15 ± 7.12 | 0.56 |
| Fasting C-peptide (ng/ml) | 2.390 ± 0.479 | 2.160 ± 0.494 | 0.76 |
| Postprandial C-peptide (2h, ng/ml) | 4.692 ± 0.820 | 4.157 ± 0.872 | 0.68 |

Continuous variables are shown as the means (SD) and categorical variables as indicated. HbA1c of 7.436% converts to 58 mmol/mol, 7.800 % to 62 mmol/mol.
